# C-Phycocyanin and Phycocyanobilin as a Novel Adjuvant in Hepatitis B Vaccine

**DOI:** 10.5812/ijpr-147060

**Published:** 2024-11-09

**Authors:** Nargess Abdali, Reza Tabaripour, Solaleh Javadi, Mehrab Nasirikenari, Mehdi Birjandi, Vahid Siavashi, Mohammad Reza Naghavi, Zahra Hasani, Ali Ahmari, Hossein Hanifi

**Affiliations:** 1 Razi Herbal Medicines Research Center, Lorestan University of Medical Science, Khorramabad, Iran; 2 Department of Cellular and Molecular Biology, Babol Branch, Islamic Azad University, Babol, Iran; 3 Comprehensive Health Research Center, Babol Branch, Islamic Azad University, Babol, Iran; 4 Department of Biotechnology, Advanced Science and Technology, Tehran Medical Sciences, Islamic Azad University, Tehran, Iran; 5 North Research Center, Pasteur Institute of Iran, Amol, Iran; 6 Nutritional Health Research Center, School of Health and Nutrition, Lorestan University of Medical Sciences, Khorramabad, Iran; 7 Department of Clinical Pathology, Faculty of Veterinary Medicine, University of Tehran, Tehran, Iran; 8 Agricultural and Natural Resources College, University of Tehran, Iran; 9 Department of Microbial Biotechnology, Amol University of Special Modern Technologies, Amol, Iran; 10 Department of Biotechnology, School of Chemical Engineering, College of Engineering, University of Tehran, Tehran, Iran

**Keywords:** Adjuvant, C-phycocyanin, Phycocyanobilin, HBs, Vaccine

## Abstract

**Background:**

Vaccine adjuvants are components that enhance immune responses to an antigen. Given the importance of adjuvants, research on novel adjuvants with higher efficacy and fewer adverse effects remains crucial. *Spirulina* (*Arthrospira* sp.), an aqueous, photosynthetic, filamentous, spiral, multicellular microalga also classified as a cyanobacterium, is well known for its high protein content, vitamins, essential fatty acids, and amino acids. C-phycocyanin (C-PC) is one of the most significant proteins in *Spirulina*.

**Objectives:**

This study aimed to investigate the adjuvant capabilities of three *Spirulina*-derived substances—*Spirulina* extract, C-phycocyanin (C-PC), and phycocyanobilin (PCB)—in conjunction with the Hepatitis B surface antigen (HBsAg).

**Methods:**

Vaccine groups received the vaccine and adjuvants three times at two-week intervals, administered either orally or by injection in encapsulated or naked forms. To use the injectable form while preventing antigenic effects from the C-PC protein portion, the PCB portion was isolated and used as an injectable adjuvant.

**Results:**

The highest levels of interferon gamma (IFN-γ) and interleukin 4 (IL-4) stimulation were observed in the naked PCB form with the vaccine. In both oral and injectable forms of PCB and C-PC, results indicated an increased expression of Hepatitis B surface antibodies (HBsAb) in response to the antigen. The absence of a significant difference between C-PC and *Spirulina* extract in oral form suggested that the adjuvant effect of this microalga was primarily due to the C-PC compound. Additionally, the injectable form of PCB led to the highest HBsAb expression level. This enhancement of the humoral immune response indicated that these compounds have potential as adjuvants in both oral and injectable forms.

**Conclusions:**

These findings suggest the potential for improved Hepatitis B vaccine efficacy with this novel adjuvant, paving the way for further evaluation with other vaccines.

## 1. Background

Vaccination is considered one of the greatest achievements of modern medicine (1). According to the World Health Organization (WHO), prior to the COVID-19 pandemic, vaccinations saved approximately 2.5 million lives annually (2). Over time, numerous studies and efforts have been dedicated to increasing vaccine effectiveness, including the incorporation of various substances known as adjuvants as primary components of vaccines (3).

Historical development of adjuvants: In 1920, Gaston Ramon, a veterinarian at the Pasteur Institute, first introduced the term “adjuvant.” He observed that horses receiving the diphtheria vaccine developed higher antibody titers when inflammatory abscesses formed at the injection site (4). Ramon demonstrated that modifying the inactivated diphtheria toxin with various chemicals induced inflammation at the injection site, enhancing antibody expression in response to the vaccine (5). Since the 1920s, numerous additional vaccines with adjuvants have been introduced (4).

Challenges with current adjuvants: Since the immunological response of a subunit of the whole microorganism, despite its specificity, is often lower than that of the whole microorganism, the need for adjuvants, especially in subunit vaccines, has become more apparent with the rise of recombinant vaccine production (6). Ideal characteristics of a potential adjuvant include safety (minimal adverse effects), neutrality, biodegradability, a long-lasting immune response, a defined half-life, economic feasibility, and minimal environmental impact (7-9). In designing and producing a vaccine, the adjuvant type is as crucial as the structure and quantity of the antigen to elicit a robust and desirable immune response (10). Other key factors when selecting an adjuvant include formulation, vaccine administration method, timing, dose volume, antigen structure, host species, interspecies variation, and the target organism’s immune system (11).

Alum is the most commonly used adjuvant, known for stimulating potent humoral immune responses (12). However, it has limitations, such as inadequate stimulation of cellular immunity, potential side effects like fever, chills, and neurological issues, and varying efficacy among individuals (13, 14). A subset of the population, termed the “non-responsive population,” does not always respond well to vaccinations. For instance, 7.5% of individuals aged 18 - 59 and 11.7% of those over 60 do not respond to the COVID-19 vaccine (15). Additionally, 10% - 15% of people who receive the Hepatitis B vaccine do not mount an adequate immune response; thus, altering the vaccine formulation, particularly by modifying the adjuvant, could enhance efficacy in these groups (13, 16, 17). Given this context and the essential role adjuvants play in vaccination, it can be argued that adjuvants are as important as, if not more than, the antigen itself.

**Figure 1.**
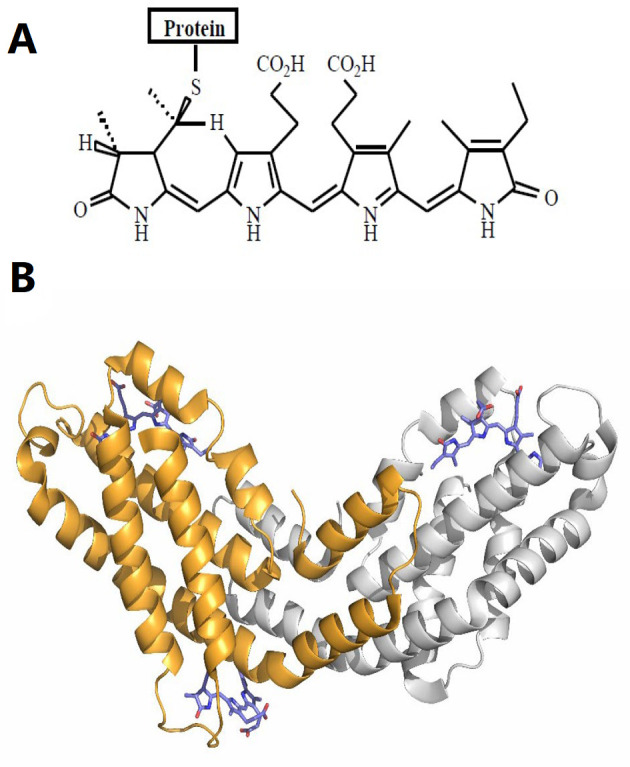
A, chemical structure of the bilin chromophores in phycocyanin (24); B, tertiary structure unit of C-phycocyanin (C-PC) from Spirulina platensis. The alpha subunit of C-PC is shown in gray, the beta subunit in orange, and phycocyanobilin (PCB) in purple stick representation (PDB ID: 1gh0).

*Spirulina* and C-phycocyanin as potential adjuvants: Researchers in this field are continually exploring novel adjuvants with greater efficacy and fewer adverse effects. *Spirulina* is well-known for its high protein content, vitamins, essential fatty acids, and amino acids (18). Notably, 55% - 70% of its dry weight is composed of protein (18-20). Phycocyanins, or phycobiliproteins (PBPs), are proteins derived from few species especially cyanobacteria, with C-phycocyanin (C-PC) being a type of PBP (21). Figure 1A and B show that the core component of PBPs is phycocyanobilin (PCB) or bilins (chromophores) (22, 23).

A noteworthy point regarding the genus *Spirulina*: While the term “*Spirulina*” is commonly used to describe this biomass, it actually refers to a taxonomic misclassification introduced in the reprint of Geitler's book (1925 - 1932), specifically for two commercial and scientific species, *Arthrospira* *platensis* and *A. maxima*. The presence of gamma linoleic acid (GLA) in *Arthrospira*, its absence in *Spirulina* (a chemotaxonomic feature), and differences in 16S rRNA sequences are key distinguishing factors (21, 25-27).

Both *Spirulina* and C-PC are classified as "Generally Recognized as Safe" (GRAS) by the US Food and Drug Administration (FDA). Except for infants, a daily dose of 250 mg of C-PC is deemed safe (28, 29). Given its beneficial properties and FDA approval—including anticancer, analgesic, and anti-inflammatory effects—C-PC shows promise as a novel adjuvant (29-32).

History of *Spirulina* and phycocyanins as adjuvants: Limited sources discuss the adjuvant use of *Spirulina*, particularly its phycocyanins. Adjuvants have typically been utilized to address cancer, diabetes, and blood lipid levels. The term "adjuvant" has been suggested for phycocyanins when used alongside medications other than vaccines (33-35). However, studies specifically involving phycocyanins and *Spirulina* in the context of adjuvants, antibodies, immune factors, and vaccines remain few (36-38).

In a study by Hirahashi et al., 12 healthy male participants received 50 mL of orally-administered *Spirulina* extract daily. Although data indicated that *Spirulina* directly or indirectly influenced natural killer (NK) cells on myeloid lineages, the study assessed only the effects of the *Spirulina* extract as a whole (36). In a 2014 review, Barzegari et al. noted the presence of substances within *Spirulina* with potential adjuvant properties, suggesting there may be other adjuvant components in *Spirulina* aside from phycocyanin (37). The first direct study of the pure composition of phycocyanin combined with antigen was conducted by Nemoto-Kawamura et al. They examined phycocyanin’s effects on immune responses and mucosal and systemic allergic inflammation in both C3H/HeN and BALB/c mice (38). Ovalbumin microparticles were used as an antigen twice weekly to enhance the immune response of mice two weeks after phycocyanin administration. Immunoglobulin antibodies IgA, IgG1, and IgE were quantified using enzyme-linked immunosorbent assay (ELISA) (38). In mice administered phycocyanin for six weeks, IgA antibody levels increased in Peyer’s patches, intestinal mucosa, mesenteric lymph nodes, and spleen cells. By the eighth week, phycocyanin reduced serum IgG1 and IgE antibody levels. After six weeks, inflammation in the small intestine was significantly reduced. Their findings suggested that phycocyanin could strengthen the immune system's defense against infectious diseases by maintaining mucosal immune function and reducing allergic inflammation through suppression of IgE antibodies against specific antigens. However, the exact form of phycocyanin used in the study was unclear (38).

## 2. Objectives

This current study represented the first evaluation of the adjuvant properties of purified C-PC and PCB from *Spirulina*. The assessment was conducted using both oral and injectable administration methods. To utilize C-PC in injectable form while avoiding the allergic and antigenic effects of its protein component, the PCB portion was isolated and used as the injectable adjuvant. Additionally, to protect the "naked form" of PCB, liposome-encapsulated PCB was also evaluated in a separate group. All forms of the adjuvant were tested alongside the Hepatitis B vaccine in BALB/c mice, a standard animal model for immunological studies. 

## 3. Methods

### 3.1. Cultivation of Spirulina

*Arthrospiraplatensis* (*Spirulina*) was cultured using salt medium (SM) (39) under controlled environmental conditions: pH range of 8 - 11, light intensity between 1000 - 3000 lux, temperature range of 25 - 32°C, and aeration for 15 minutes twice daily.

### 3.2. Confirmatory Tests of the Genus Arthrospira 

To verify the genus authenticity, 16S rRNA molecular testing and fatty acid profile analysis were conducted, addressing the taxonomic misclassification discussed in the introduction. *Spirulina* DNA was extracted using an optimal boiling method, followed by PCR amplification with forward (AGAGTTTGATCMTGGCTCAG) and reverse (GGTTACCTTGTTACGACTT) primers. The PCR program included 35 cycles: Denaturation for 45 seconds at 94°C, annealing for 1 minute at 57°C, and DNA extension for 1 minute at 72°C. The fatty acid profile was analyzed following the ISIRI-13126-1 standard (40, 41).

### 3.3. Purification of C-phycocyanin

To achieve food-grade purification of C-PC, *Spirulina* was subjected to five freeze-thaw cycles in a 0.05 M Na-phosphate buffer (pH = 7.0). This was followed by sonication at 48 kHz for 5 minutes at room temperature using an ultrasonic bath (CD-4820 model), and then centrifugation at 14000 rpm for 20 minutes at 4°C. The precipitate was discarded, and the supernatant was collected for use (42).

The concentration and purity of C-PC were determined with a UV/VIS spectrophotometer (T80+ model) using the following formulas (43, 44):


$$Concentration\;\left(\frac{mg}{ml}\right)=\;\frac{\left(A_{620}(0.474-\times A_{652}\right)}{5.34}$$

$$Purity=\frac{A_{620}}{A_{280}}$$

For confirmatory analysis, HPLC was performed based on PCB with a C18 column, a 590-nm detector, a flow rate of 5 mL/min, and a water/methanol mobile phase. This analysis was conducted at the Nanoscience and Nanotechnology Institute (INST) of Sharif University. The HPLC standard was obtained from Sigma (P2172).

### 3.4. Cleaving of Phycocyanobilin

The dried and purified C-PC was dissolved in 100% methanol, left to stand for 15 minutes, and then centrifuged at 3000 rpm for 15 minutes. To remove unbound tetrapyrroles and contaminants, the sample was washed multiple times, and the supernatant was discarded. In a 500-mL distillation flask connected to a condenser, 100 mL of methanol was added per 1 g of C-PC. Stirring was conducted in a flask placed in a silicone oil bath at a temperature 10°C above the methanol boiling point. Increasing the reaction temperature raised the reaction rate, with the optimal temperature around 75°C. Temperatures higher than this did not increase efficiency; instead, they reduced it. The reaction was stopped after 16 hours by cooling the flask in an ice bath for 15 minutes. In the conventional reflux method, a 16-hour reaction time is ideal for maximum efficiency, as extending it does not further increase yield. The supernatant was then filtered through filter paper. The deep blue solution was transferred to a separating funnel, and distilled water was added in a 2: 1 ratio, followed by a few milliliters of chloroform. The funnel was sealed, thoroughly mixed, and the chloroform phase was separated. Using a freeze dryer, the chloroform phase containing PCB was evaporated and dried (23). To evaluate PCB, the absorbance ratio at A_620_/A_280_ is commonly used, ensuring A_280_ is 0 or near it (45).

### 3.5. Liposomal Encapsulation of Phycocyanobilin

To encapsulate PCB in liposomes, DOTAP Liposomal Transfection Reagent (Sigma 144189731) was used. A 4.1 mg sample of PCB was combined with 410 µL of TE buffer and vortexed. Next, 820 µL of DOTAP, diluted in HEPES-buffered saline at a 3: 7 ratio, was added to the mixture and gently stirred. After incubating at room temperature for 15 minutes, the preparation was stored at 4°C until use.

### 3.6. Animal Testing Phase

Inbred BALB/c male mice, aged 6 - 7 weeks and weighing 20 - 25 grams, were selected for this study and housed individually in polycarbonate cages with unrestricted access to food and water. The mice were kept at a constant temperature of 22.2°C and a humidity level of 55%, and they underwent a 12-hour fast prior to treatment. This study examined the impact of the adjuvant on enhancing the immune response, specifically the expression of Hepatitis B antibody, compared to the negative control (distilled water) and positive control (a traditional vaccine produced at the Pasteur Institute of Iran; IRC:1228070570, batch No. 97BD221). The vaccine was administered intraperitoneally. The vaccine groups received the standard vaccine with adjuvant three times at two-week intervals, containing Hepatitis B surface antigen (HBsAg) and alum. Novel adjuvants (C-PC and PCB) were administered to the test groups either orally, intravenously, or both, in either encapsulated or naked form. Tables 1 and 2 provide all pertinent information, abbreviations, and classifications of animal types and administered doses. Humoral immune responses were evaluated two weeks after the final treatment (third dose).

**Table 1.** Average Results of Studied Factors in Groups Receiving Injectable Adjuvants

No.	Group Name	Abbreviation	BILB/cNo.	Consumption Dose		The Average Range of the Studied Factors				
				Adjuvants	Vaccine, µg	HBsAb, IU/mL	IL-4, pg/mL	IFN-γ, pg/mL	ALT, U/l	AST, U/l
**1**	HBsAg vaccine ^a^ + liposome adjuvant ^b^	Vac. + liposome	3	150 µg liposome used beside vaccine	10	740.83	3.72	12.18	62.00	215.33
**2**	HBsAg vaccine ^a^ +new adjuvant phycocyanobilin	I.PCB + Vac.	3	500 µg injectable PCB used beside vaccine	10	1349.67	11.08	25.41	70.67	263.67
**3**	HBsAg vaccine ^a^ + liposomal encapsulated phycocyanobilin	Vac. + liposomal I.PCB	3	500 µg injectable PCB encapsulated with 150 µg liposome used beside vaccine	10	1102.33	6.47	20.02	69.33	243.33
**4**	Control -	I.C -	3	-	-	132.93	2.64	10.70	86.33	292.33
**5**	Control + (HBsAg vaccine ^a^)	I.C +	3	-	10	1118.67	8.23	19.33	47.00	171.67
	Total		15	-	888.89	6.43	17.53	67.07	237.27	

Abbreviations: HBsAb, Hepatitis B surface antibody; IL-4, interleukin 4; IFN-γ, interferon gamma; ALT, alanine aminotransferase; AST, aspartate aminotransferase.

^a^ This is a routine vaccine that includes alum as an adjuvant.

^b^ A liposome was used as an adjuvant in the "HBsAg vaccine + liposomal as an adjuvant" group to isolate and remove the effect of liposomes, essentially serving as a control+ group.

**Table 2.** Average Results of Studied Factors in Groups Receiving Oral Adjuvants

No.	Group Name	Abbreviation	BILB/cNo.	Consumption Dose		The Average Range of the Studied Factors				
				Adjuvants	Vaccine, µg	HBsAb, IU/mL	IL-4, pg/mL	IFN-γ, pg/mL	ALT, U/l	AST, U/l
**1**	*Spirulina*/*Arthrospiraplatensis* extraction	O.SE	4	1000 µg oral SE, 2 times weekly for 8 weeks	-	33.00	146.80	760.59	136.50	304.75
**2**	*Spirulina*/ *Arthrospiraplatensis* extraction + HBs vaccine ^a^	O.SE ^+^ Vac	6	1000 µg oral SE 2 times weekly for 8 weeks	**10 µg**	493.33	370.24	821.96	62.17	102.17
**3**	New adjuvant C-phycocyanin	O. C-PC	6	500 µg oral C-PC, 2 times weekly for 8 weeks	-	52.00	143.13	397.25	106.00	293.50
**4**	New adjuvant c-phycocyanin + HBs vaccine ^a^	O.C-PC ^+^ Vac	4	500 µg oral C-PC/2 times weekly for 8 weeks	10 **µg**	424.75	382.13	778.82	72.50	210.00
**5**	C^-1^ (sterile water)	O.C^-^ w	3	-	-	17.00	105.36	395.29	59.00	114.67
**6**	C^+1^ (vaccine ^a^ + sterile water)	O.C^+^ w	3	-	10 **µg**	190.00	533.80	1254.90	212.00	278.33
**7**	C^-2^ (nothing)	O.C^-^	3	-	-	10.00	134.24	494.12	58.00	155.67
**8**	C^+2^ (vaccine ^a^)	O.C^+^	3	-	10 **µg**	212.00	356.46	2242.35	65.33	202.33
	Total/mean		32	-	-	199.69	268.30	832.28	94.63	208.94

Abbreviations: HBsAb, Hepatitis B surface antibody; IL-4, interleukin 4; IFN-γ, interferon gamma; ALT, alanine aminotransferase; AST, aspartate aminotransferase.

^a^ This is a routine vaccine that includes alum as an adjuvant.

### 3.7. ELISA and Biochemical Method 

ALT and AST levels were measured using an autoanalyzer. Additional immunological factors, including IL-4 (Cat No: M4000B, R&D™, USA), IFN-gamma (Cat No: MIF00, R&D™, USA), and HBsAb (Cat No: PT-ANTI HBS-96, Pishtazteb™, Iran), were assessed using the ELISA technique according to the kit instructions.

### 3.8. Statistical Analysis

The statistical analysis was conducted based on the averages derived from all data. Means of the samples were compared using SPSS version 22 (SPSS Inc., Chicago, IL, USA). Data normality was evaluated with the Shapiro-Wilks test; upon confirming normality, a one-way analysis of variance was used for group comparisons. P-values less than 0.05 were considered statistically significant if the 95% confidence interval (CI) was met.

## 4. Results 

### 4.1. Verification of Genus and Species

As previously noted, analyzing the fatty acid profile and genetic differentiation in 16S rRNA sequences are two essential factors for identification and differentiation from a chemotaxonomy perspective (21, 25-27). Due to the importance of the project, these two confirmation tests were performed to verify the accuracy of the genus and species.

In our study, the presence of gamma-linolenic acid (GLA) at a concentration of 0.848% was identified through fatty acid profile analysis, confirming the genus as *Arthrospira*. The presence of GLA in *Arthrospira* and its absence in *Spirulina* are critical identifiers from a chemotaxonomy perspective.

As shown in Appendix 1 in Supplementary File, the BLAST analysis of the strain sequence in NCBI indicates that our sequence aligns with the registered strain sequence in NCBI (accession number AP026945.1), which corresponds to the complete genome of *Arthrospira* *platensis*, with 99.29% identity and an E-value of 0.0.

### 4.2. Chemical Analysis for Purified C-PC Confirmation

Appendix 2 in Supplementary File presents the concentration and purity outcomes of purified C-PC, as confirmed by the HPLC graph. UV spectrophotometry indicated a purity level of 1.94.

According to Rito-Palomares et al., various purity levels for C-PC include 0.7 for food grade, 3.9 for reactive grade, and greater than 4.0 for analytical grade (44). Although food-grade purity suffices for oral adjuvant studies, our purification result (1.94) indicates a higher, favorable grade. Given that C-PC includes a protein and a bilin portion, to develop it as an injectable adjuvant without the antigenic effect of the protein part, the PCB component (bilin) was isolated and purified. The objective was to evaluate PCB's adjuvant properties as an injectable form of C-PC and to demonstrate that PCB alone can provide similar adjuvant efficacy as C-PC.

### 4.3. Animal Test Results

The average results for each group are presented in Tables 1 and 2. Comparisons of the averages between injectable and oral adjuvants are shown in Appendices 3 and 4 in Supplementary File, respectively.

**Figure 2.**
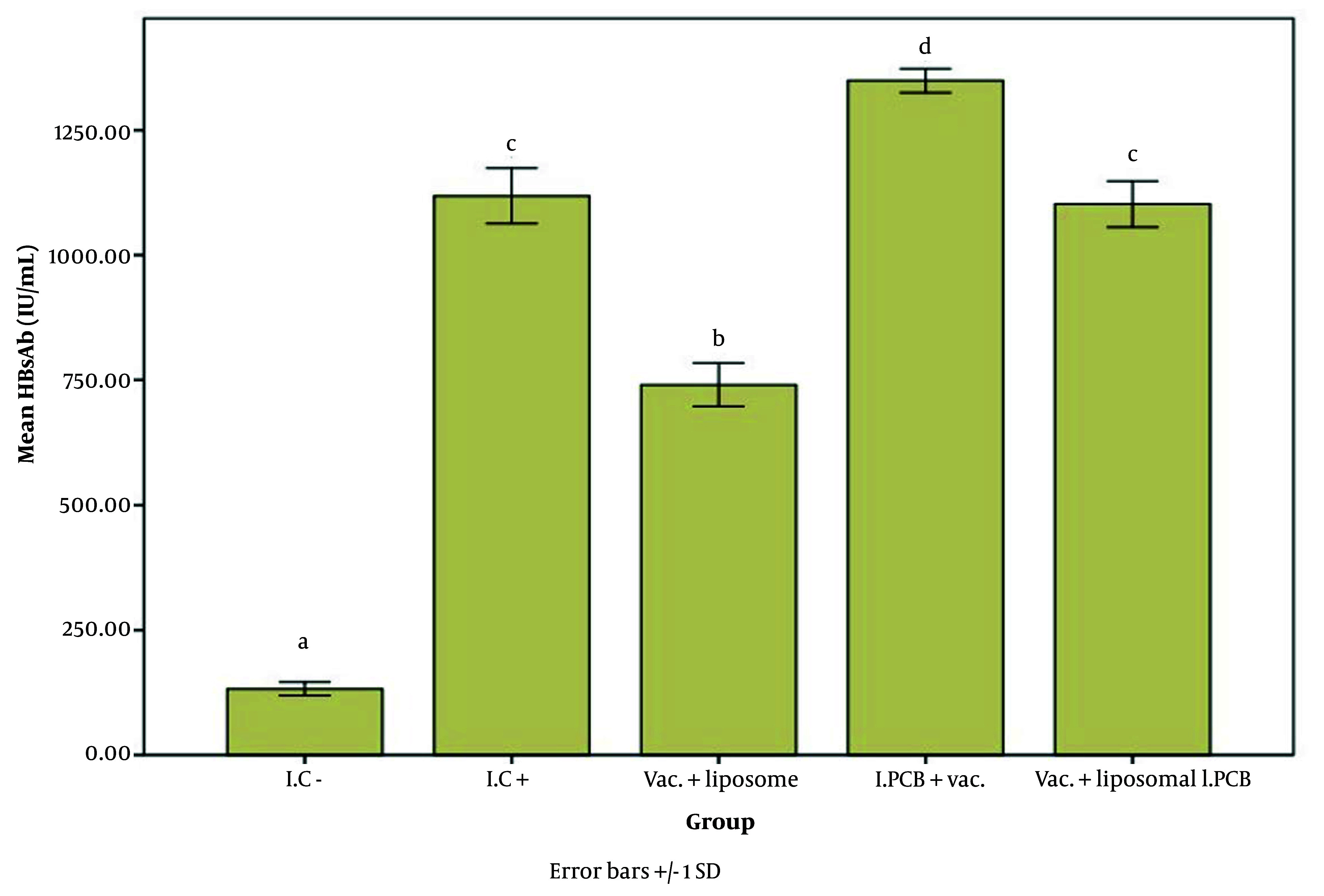
Comparison of the average level of Hepatitis B surface antibody (HBsAb) expression in response to Hepatitis B surface antigen (HBsAg) among different groups receiving the injectable adjuvant (IC^-^: 132.93, IC^+^: 1118.67, Vac. Liposome: 740.83, I.PCB+Vac.: 1349.67, & Vac. + Liposomal I.PCB: 1102.33; P = 0.000). Statistically, treatments are categorized into four distinct groups with significant differences (a - d).

**Figure 3.**
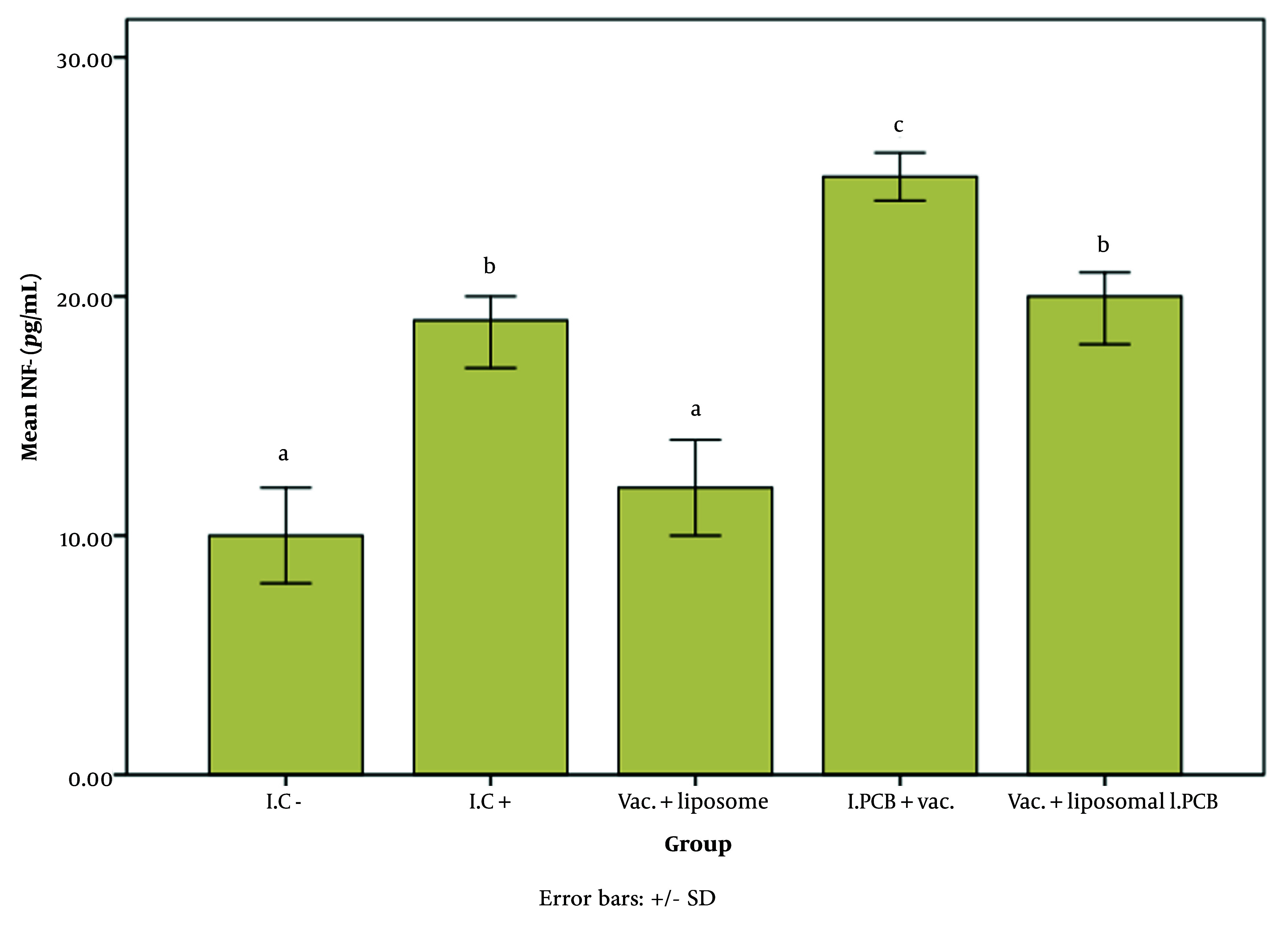
Comparison of the average level of interferon gamma (IFN-γ) expression among different groups receiving the injectable adjuvant (IC^-^: 10.70, IC^+^: 19.33, Vac. Liposome: 12.18, I.PCB+Vac.: 25.41, & Vac. + Liposomal I.PCB: 20.02; P = 0.000). Statistically, treatments are categorized into three distinct groups with significant differences (a - d).

**Figure 4.**
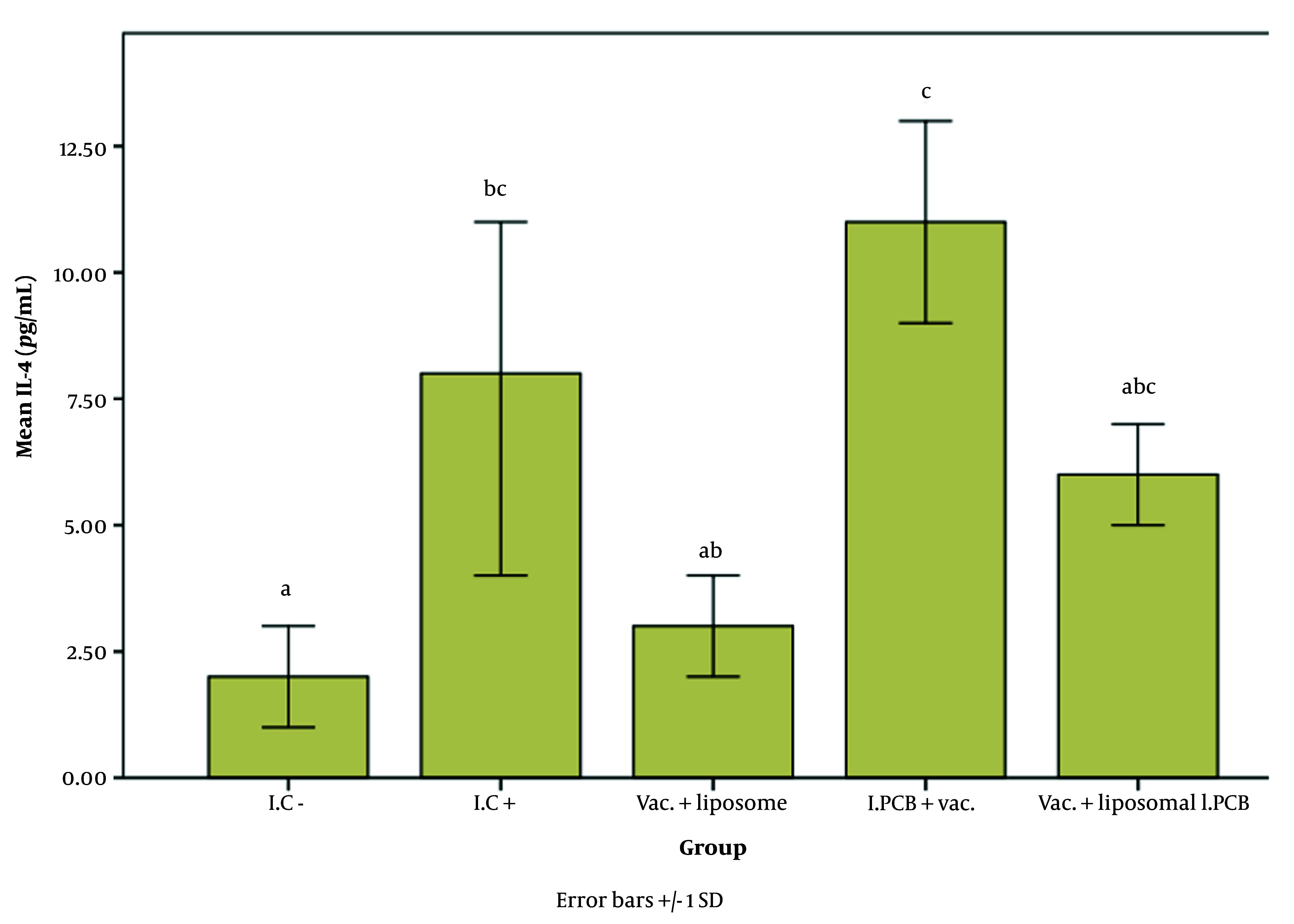
Comparison of the average interleukin 4 (IL-4) expression level among different groups receiving the injectable adjuvant (IC^-^: 2.64, IC^+^: 8.23, Vac. Liposome: 3.72, I.PCB+Vac.: 11.08, & Vac. + Liposomal I.PCB: 6.47; P = 0.003). Although the treatments are classified into three main groups (a, b, and c), there are significant differences displayed across four subgroups (a, c, abB, and bcC). It can be observed that the "b" subgroups (ab, bc, and abc) and the "c" subgroups (c, bcC, and abcBC) show no significant differences within their respective subgroups.

Figures 2 - 4 illustrate the effect of the PCB adjuvant, used as an injectable form of C-PC, on HBsAb, IFN-γ, and IL-4, respectively.

**Figure 5.**
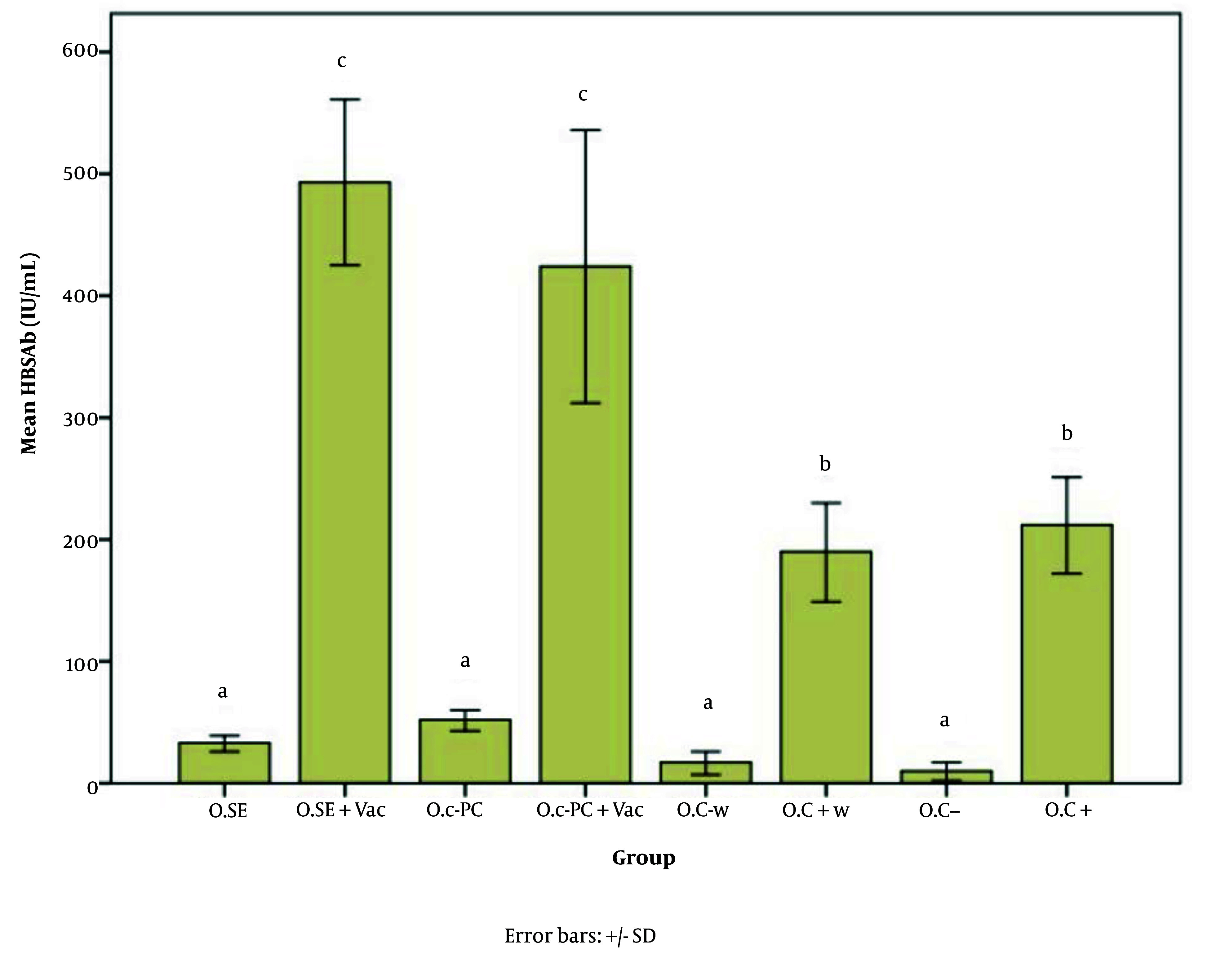
Hepatitis B surface antibody (HBsAb) expression in response to the new oral adjuvant (O.SE: 33.00, O.SE + Vac.: 493.33, O.C-PC: 52.00, O.C-PC + Vac.: 424.75, O.C-w: 17.00, O.C + w: 190.00, O.C-: 10.00, & O.C+: 212.00; P = 0.000). Statistically, the treatments are divided into three distinct groups with significant differences (a - c).

**Figure 6.**
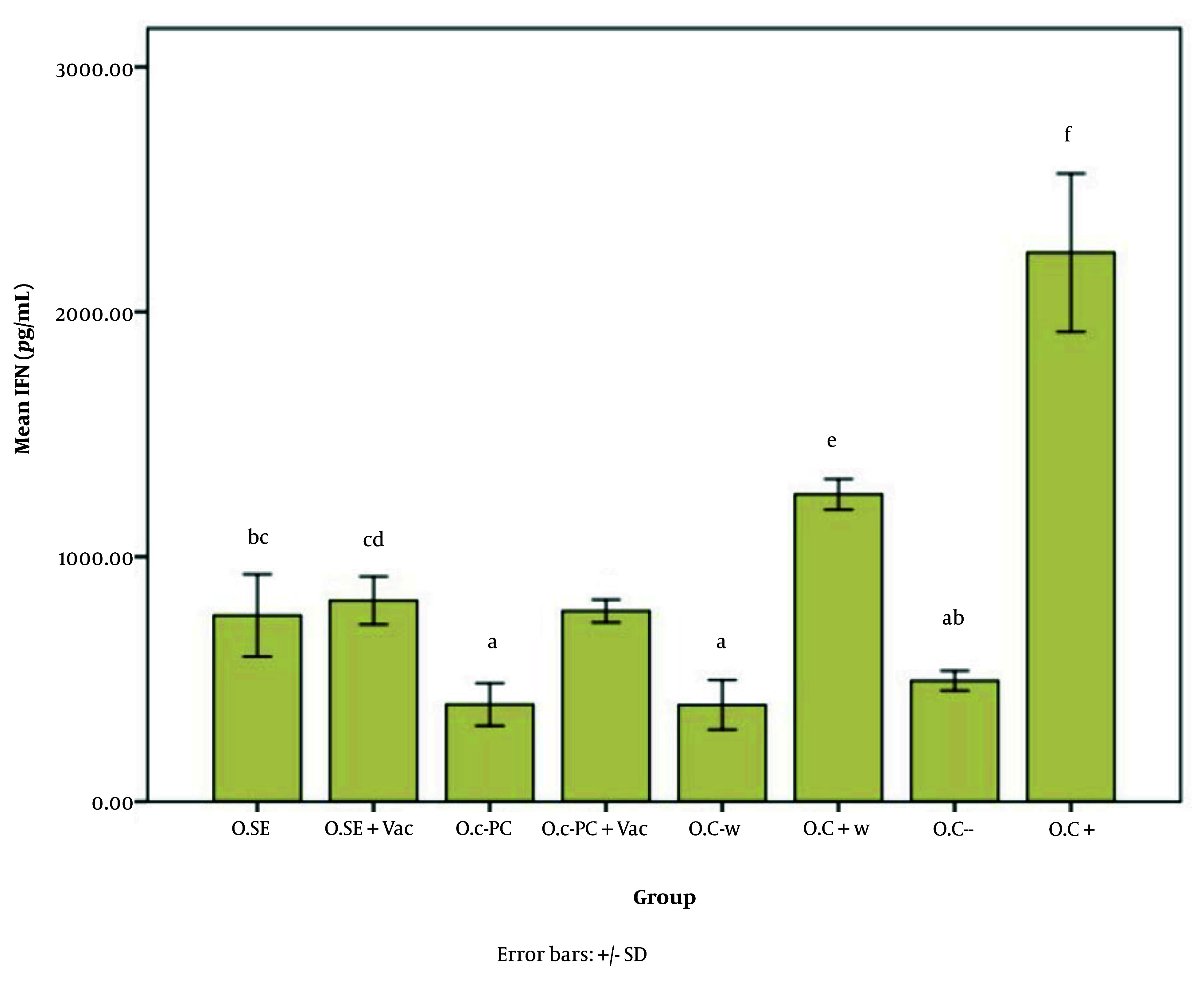
Interferon gamma (IFN-γ) expression level in response to the oral adjuvant (O.SE: 760.59, O.SE + Vac.: 821.96, O.C-PC: 397.25, O.C-PC + Vac.: 778.82, O.C-w: 395.29, O.C + w: 1254.90, O.C-: 494.12, & O.C+: 2242.35; P = 0.000). Although categorized into six main groups (a, b, c, d, e, and f), significant differences appear across the following groups: a, ab, bc, cd, e, & f. The "b" subgroups (BC & AB) and "c" subgroups (BC & CD) show no significant differences within their own groups.

**Figure 7.**
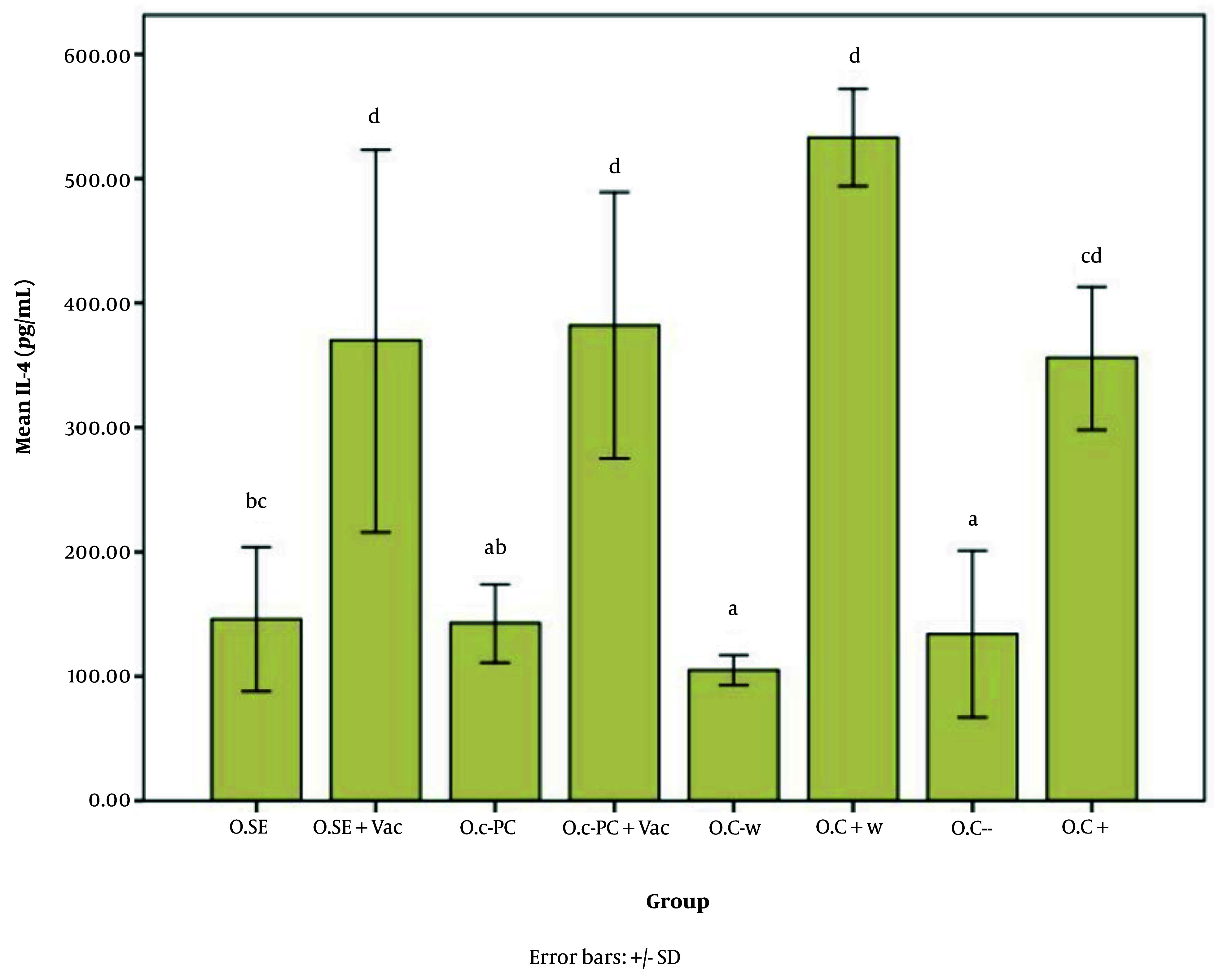
Interleukin 4 (IL-4) expression level in response to the oral adjuvant (O.SE: 146.80, O.SE + Vac.: 370.24, O.C-PC: 143.13, O.C-PC + Vac.: 382.13, O.C-w: 105.36, O.C + w: 533.80, O.C-: 134.24, & O.C+: 356.46; P = 0.000). While treatments are grouped into four main categories (a, b, c, and d), significant differences appear across groups a, ab, bc, cd,and d. The "b" subgroups (bc and ab) and "c" subgroups (bc and cd) show no significant differences within their respective groups.

Figures 5 - 7 illustrate the effect of S.E. (*Spirulina* extract) and purified C-PC (C-phycocyanin) adjuvants in various oral forms on HBsAb, IFN-γ, and IL-4, respectively.

## 5. Discussion

### 5.1. Examining the Results of Adjuvant Use in Injectable Form

The level of Hepatitis B surface antibody (HBsAb) expression varied significantly among different groups, as shown in Figure 2. At a 95% confidence interval, the expression level in the I.C^-^ group was significantly different from all groups receiving the vaccine (P = 0.000).

Comparing the test groups with the positive controls, the I.PCB + Vac. group exhibited a significantly higher level of antibody expression than all other groups. As shown in Table 1, the I.C^+^ group had an average antibody response of 1118.66 units, whereas the I.PCB+ Vac. group achieved an average expression level of 1349.66 units, indicating an approximately 1.2-fold increase.

When comparing the Vac. + liposomal I.PCB group to the I.C^+^ group, no notable differences were found, and their performance was classified in the same category. Thus, it appears that the liposomal encapsulated PCB adjuvant may have a reducing or inhibitory effect on the efficacy of the PCB injectable adjuvant. To assess the potential impact of the liposome alone on antibody expression in the immune response to the vaccine, a comparison was made between the Vac. + liposome and I.C^+^ groups. Results indicated that the liposome did not display any adjuvant properties with this type of vaccine in the current study. In fact, antibody expression decreased significantly compared to the I.C^+^ group, suggesting a lowered level of immune stimulation in the presence of liposomes.

Comparing the Vac. + liposomal I.PCB group to the Vac. + liposome group is particularly noteworthy, as it highlights the effectiveness of the PCB adjuvant even in the presence of liposomes, which were shown to inhibit HBsAb production in this study. Adding PCB to liposomes seemed to mitigate the negative effects of the liposomes.

Adjuvants are essential components of vaccines. Although widely used, the mechanisms underlying their action are not yet fully understood. However, as insights into how the innate immune response regulates the antigen-specific response have grown, the mechanisms of adjuvants are gradually being clarified (46). Adjuvants can be divided into two primary categories: Immune stimulators and delivery systems (such as liposomes) (47). Liposomes consist of a spherical phospholipid bilayer surrounding equal numbers of aqueous compartments (48). In this study, liposomes were used for their delivery capability to preserve PCB, as its protein cover had to be removed for injection. Although liposomes are commonly used as adjuvants for delivery, a modified form, ALF, introduced in the 1980s, also functions as an immunostimulant (49). While some studies suggest that vaccines containing liposome-localized antigens may have a greater effect (50), in this case, the adjuvant PCB was embedded in the liposome rather than the antigen to protect it. The results showed that the liposome not only did not function as a delivery adjuvant here but also inhibited the action of PCB. Theoretically, it is possible that liposomes either prevented the release of PCB, led to a timing mismatch in PCB release relative to antigen action, produced an inhibitory response, or neutralized the PCB effect.

As shown in Figure 3, the level of IFN-γ in the I.PCB + Vac. group was significantly higher than in other groups (P = 0.000). According to Table 1, the increase in IFN-γ was approximately 2.5 times higher than in the I.C^-^ group.

The expression level of interferon gamma (IFN-γ) in the Vac. + liposome group did not differ significantly from that in the I.C^-^ group. While the alum adjuvant in the vaccine likely contributed to the increase in IFN-γ in the I.C^+^ group compared to I.C^-^, the presence of liposomes in the Vac. + liposome group appears to have an inhibitory or reducing effect on this factor once again.

Despite the presence of PCB, the Vac. + liposomal I.PCB group remains at the same level as the I.C^+^ group, reaffirming the liposome's inhibitory effect on the performance of the new adjuvant.

According to Figure 4, the results for the interleukin 4 (IL-4) factor are as follows: The I.C^+^ group showed a significant difference compared to the I.C^–^ group. The groups were classified into three main categories in terms of IL-4 expression: Class a: I.C^-^, Vac. + liposome, and Vac. + liposomal I.PCB; Class b: I.C^+^, Vac. + liposome, and Vac. + liposomal I.PCB; and Class c: I.C^+^, I.PCB + Vac., and Vac. + liposomal I.PCB.

The grouping of both liposome-containing groups with I.C^-^ indicates the strong inhibitory effect of the liposome on stimulating the IL-4 factor for both PCB and alum adjuvants. On the other hand, although the I.C^+^ group (i.e., the standard vaccine) was placed in category B, its proximity to the Vac. + liposome and Vac. + liposomal I.PCB groups shows a notable improvement in IL-4 expression for both liposome-containing groups compared to I.C^-^. The fact that all groups receiving the vaccine with the new adjuvant (PCB) were placed in category C (while the Vac. + liposome group was outside this category) highlights the beneficial effect of our PCB adjuvant in mitigating the limiting impact caused by the liposome. As with HBsAb and IFN-γ, the highest level of IL-4 stimulation was observed in the I.PCB + Vac. group. According to Table 1, this increase in expression compared to I.C^-^ and I.C^+^ (the standard vaccine) is approximately 4.2 times and 1.35 times, respectively.

As shown in Appendices 5 and 6 in Supplementary File, no significant changes were observed in alanine aminotransferase (ALT; P = 0.133) and aspartate aminotransferase (AST; P = 0.425) levels in the studied groups, indicating no adverse effects on liver biochemical factors.

The optimal outcome in this investigation and the use of PCB as an injectable form of the C-PC adjuvant was achieved with PCB alone. Further studies are recommended to explore the inhibitory function of liposomes.

### 5.2. Examining the Results of Oral Adjuvant Administration

Based on Figure 5, all groups that did not receive the vaccine, including O.C- w, O.C-, O.SE (oral consumption of *Spirulina* extract), and O.C-PC, are classified in category A. Both positive control groups receiving the vaccine alone (O.C+ w and O.C+) are classified in category B. Both groups that received *Spirulina* extract and pure adjuvant composition (i.e., O.SE + Vac. and O.C-PC + Vac.) are classified in category C.

The significant increase in HBsAb expression in the O.SE + Vac. and O.C-PC + Vac. groups is at least 2.3 and 2 times the expression ratio of the positive control groups (O.C+ w and O.C+), respectively (P = 0.000). 

Although there was no significant difference between the O.SE + Vac. and O.C-PC + Vac. groups, the slightly greater adjuvant effect observed in the O.SE + Vac. group may be due to the presence of additional stimulatory compounds in *Spirulina* besides C-PC. As mentioned by Barzegari et al., *Spirulina* has immunostimulatory effects, which they attribute to CpG-rich oligodeoxynucleotides that act as adjuvants (35, 37, 51). While the minor difference between these two groups may be due to compounds naturally present in *Spirulina*, the primary adjuvant effect can still be attributed to C-PC, which originates from the PCB component of C-PC, as demonstrated by the data from the oral study on both *Spirulina* extract and the pure composition in this article.

According to Figures 6, and 7, the effects of the novel oral adjuvant on IFN-γ and IL-4 were evaluated, showing that the groups receiving the new adjuvant with the vaccines (O.C-PC + Vac. and O.SE + Vac.) differed significantly from the negative control groups (O.C- w and O.C-) (P = 0.000). These groups also showed similar impacts on ALT (P = 0.006) and AST (P = 0.000). Although significant differences were observed among the groups that received the oral adjuvant, as shown in Appendices 7 and 8 in Supplementary File, these variations did not follow any specific pattern indicative of harmful effects from C-PC. Notably, the injection groups in this study did not show any alterations in ALT or AST levels. Additionally, FDA approval and multiple studies, such as Jensen et al., have demonstrated that phycocyanin poses no adverse effects on the liver. Some studies even suggest that C-PC is currently under investigation as a treatment for fatty liver (52).

Several mechanisms have been proposed to explain how adjuvants exert their effects: (1) up-regulation of chemokines and cytokines, (2) stable release of antigen at the injection site, known as the depot effect, (3) promotion of immune cell recruitment to the injection site, (4) facilitation of antigen uptake and presentation by antigen-presenting cells (APCs), (5) activation and maturation of APCs followed by emigration into draining lymph nodes, and (6) activation of inflammasomes (53). This study focused on two cytokines to investigate the immune-stimulating mechanism, with evidence suggesting the presence of a cytokine up-regulation mechanism. Moreover, the activation of the immune response by this compound as an adjuvant has enhanced Hepatitis B antibody production.

While alum is commonly used in the HBs vaccine, it is less ideal for vaccines containing recombinant proteins or small peptides due to its inherent low immunogenicity. Alum has demonstrated a synergistic effect when combined with other adjuvants, improving their efficacy (54). Evidence from this research indicates a synergistic effect between alum and PCB, enhancing immunogenicity and HBsAb expression. This study has explored the adjuvant potential of PCB and C-PC, and further extensive studies are required to investigate their mechanisms of action and synergistic effects.

### 5.3. Conclusions 

This study examined the adjuvant properties of C-PC and PCB (as an injectable form of C-PC). The results demonstrate that both oral and injectable forms of the novel adjuvant significantly increased HBsAb expression in groups that received the vaccine alongside any form of the new adjuvant, including *Spirulina*, C-phycocyanin, and phycocyanobilin (O.SE + Vac., O.C-PC + Vac., and I.PCB + Vac. groups) (P = 0.000 and P = 0.000) compared to the positive controls. In conclusion, PCB is confirmed to be responsible for the adjuvant properties of C-PC and *Spirulina*. The potential for oral administration allows its combined use with oral medications and vaccines. On the other hand, for the injectable form, considering the complex pathway for PCB separation, examining the feasibility of synthesizing this compound becomes necessary.

It should be noted that this study was conducted using the minimum adjuvant dose, and increasing the dose in future studies is recommended. Additionally, aligning oral and injectable doses based on injection absorption efficiency and further investigation into the inhibitory functions of liposomes (including molecular interactions, encapsulation and release efficiency, and time release) are suggested.

## supplementary material

ijpr-23-1-147060-s001.pdf

## Data Availability

The dataset presented in this study is available upon request from the corresponding author during submission or after publication.
