# Supplementary material for: C-Phycocyanin and Phycocyanobilin as a Novel Adjuvant in Hepatitis B Vaccine
Source: Iran J Pharm Res. 2024 Nov 9;23(1):e147060. doi: 10.5812/ijpr-147060 (PMC11742379; doi:10.5812/ijpr-147060)
Supplement: ijpr-23-1-147060-s001.pdf [file ijpr-23-1-147060-s001.pdf]

Sequences producing significant alignments

Download ˅ Select columns ˅ Show 100

select all 100 sequences selected

GenBank Graphics Distance tree of results MSA Viewer

Description	Scientific Name	Max Score	Total Score	Query Cover	E value	Per. Ident	Acc. Len	Accession
Arthrospira platensis NIES-46 gene for 16S ribosomal RNA,...	Arthrospi...	1279	1279	99%	0.0	99.02%	1485	LC455668.1
Arthrospira platensis A 16S ribosomal RNA gene, partial seq...	Arthrospi...	1279	1279	100%	0.0	98.75%	759	MH318616.1
Arthrospira platensis YZ genome	Arthrospi...	1279	2558	98%	0.0	99.29%	6520772	CP013008.1
Arthrospira platensis NIES-39 DNA, complete genome	Arthrospi...	1279	2558	98%	0.0	99.29%	6818916	AP026945.1

Appendix 1. Blast result in NCBI.

Base	: Not Used	Calibration File	: None	Calculation	: Uncal
Scale Factor	: Not Used	Units After Scaling	: Not Used	Uncal. Response	: 0
Unretained Time	: 0.00 min	Column Length	: 50.00 mm	Column Calc.	: From Width at 50% of Height
Result Table Reports	: All Peaks	Hide ISTD Peak	: Enabled		

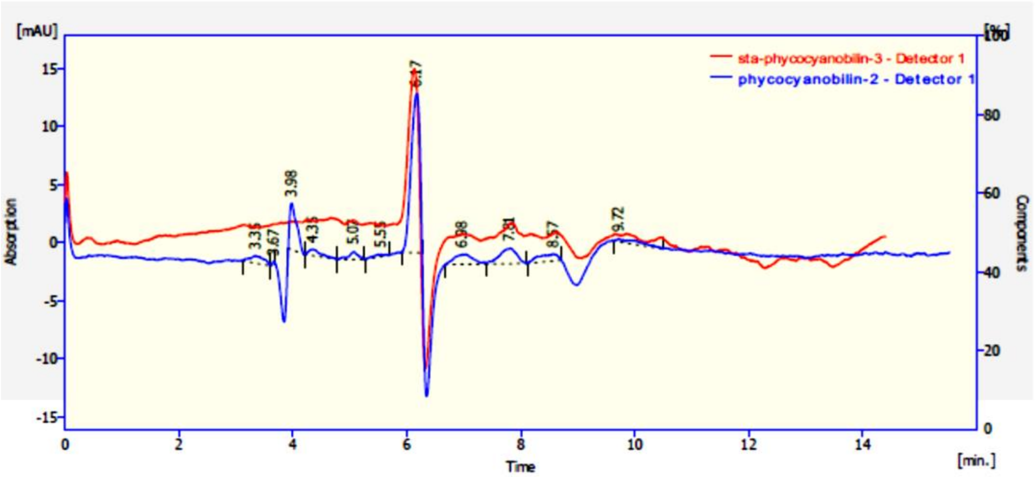

Appendix 2. HPLC peak of c-Phycocyanin (Blue peak is the sample; Red Peak is the standard).

Appendix 3. The Comparisons of the Averages of Animal Tests in the Injectable Adjuvant Form.

		Sum of squares	Df	Mean square	F	Sig.	Power of study
AST (U/l)	Between the groups	25651.600	4	6412.900	1.059	0.425	0.23

	Within the groups	60541.333	10	6054.133			
	Total	86192.933	14				
ALT (U/l)	Between the groups	2452.933	4	613.233	2.273	0.133	0.46
	Within the groups	2698.000	10	269.800			
	Total	5150.933	14				
HBsAb (IU/ml)	Between the groups	2712185.304	4	678046.326	430.998	0.000	1
	Within the groups	15732.013	10	1573.201			
	Total	2727917.317	14				
IFN-γ (*pg*/ml)	Between the groups	440.333	4	110.083	38.161	0.000	1
	Within the groups	28.847	10	2.885			
	Total	469.180	14				
IL-4 (*pg*/ml)	Between the groups	139.676	4	34.919	8.184	0.003	0.97

	Within the groups	42.665	10	4.267		
	Total	182.342	14			

Abbreviations: AST, aspartate aminotransferase; ALT, alanine aminotransferase; HBsAb, hepatitis B surface antibody; IFN-γ, interferon gamma; IL-4, interleukin 4.

Appendix 4. The Comparisons of the Averages of Animal Tests in the Oral Adjuvant Form

		Sum of squares	Df	Mean square	F	Sig.	Power of study
IL-4 (*pg*/ml)	Between the groups	635593.481	7	90799.069	11.699	0.000	1
	Within the groups	186275.852	24	7761.494			
	Total	821869.333	31				
IFN-γ (*pg*/ml)	Between the groups	8584785.525	7	1226397.932	70.542	0.000	1
	Within the groups	417248.904	24	17385.371			
	Total	9002034.429	31				
HBsAb	Between the groups	1170792.792	7	167256.113	58.995	0.000	1

(IU/ml)	Within the groups	68042.083	24	2835.087			
	Total	1238834.875	31				
ALT (U/l)	Between the groups	67806.000	7	9686.571	3.866	0.006	0.93
	Within the groups	60131.500	24	2505.479			
	Total	127937.500	31				
AST (U/l)	Between the groups	197782.125	7	28254.589	9.774	0.000	1
	Within the groups	69377.750	24	2890.740			
	Total	267159.875	31				

Abbreviations: IL-4, interleukin 4; IFN-γ, interferon gamma; HBsAb, hepatitis B surface antibody; ALT, alanine aminotransferase; AST, aspartate aminotransferase.

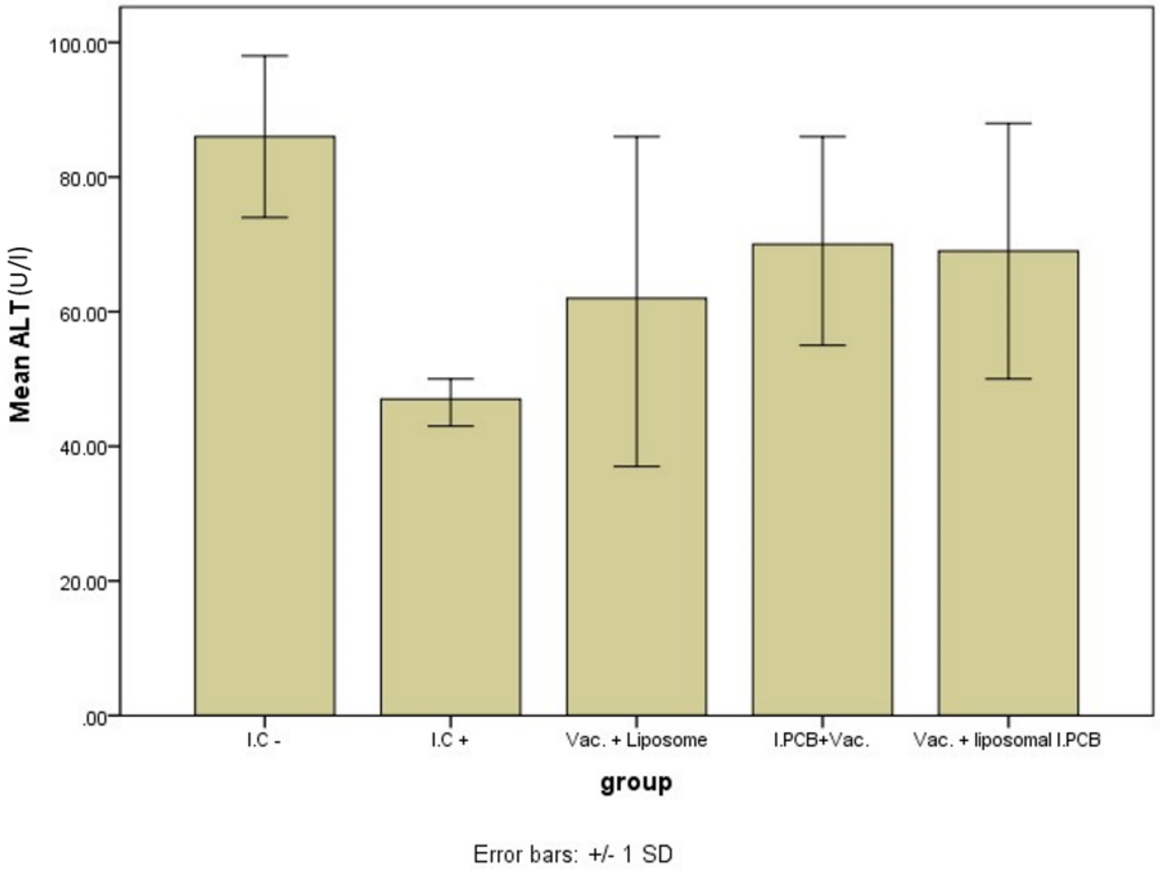

Appendix 5. Comparison of the average level of ALT changes in different groups receiving injectable adjuvant (IC-: 86.33, IC+: 47.00, Vac. Liposome: 62.00, I.PCB +Vac.: 70.67 & Vac. +Liposomal I.PCB: 69.33).

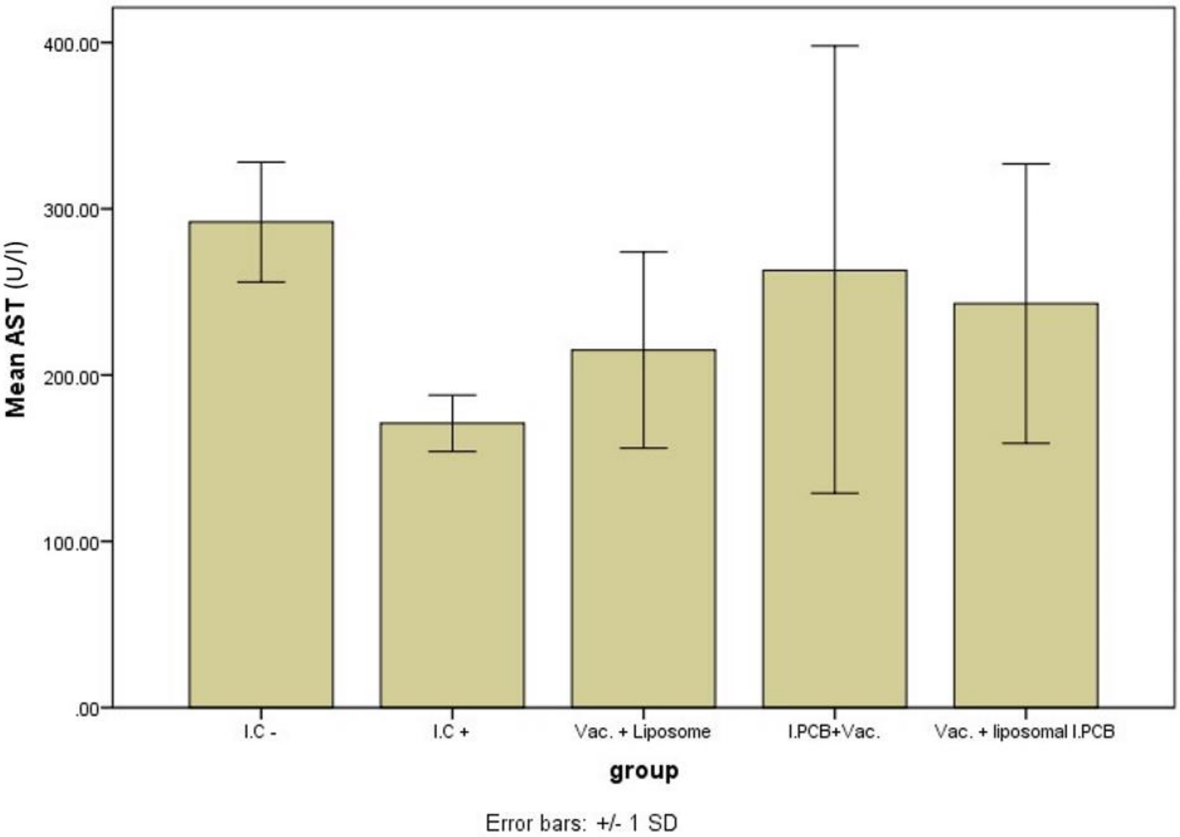

Appendix 6. Comparison of the average level of AST changes in different groups receiving injectable adjuvant (IC-: 292.33, IC+: 171.67, Vac. Liposome: 215.33, I.PCB+Vac.: 263.67 & Vac.+Liposomal I.PCB: 243.33).

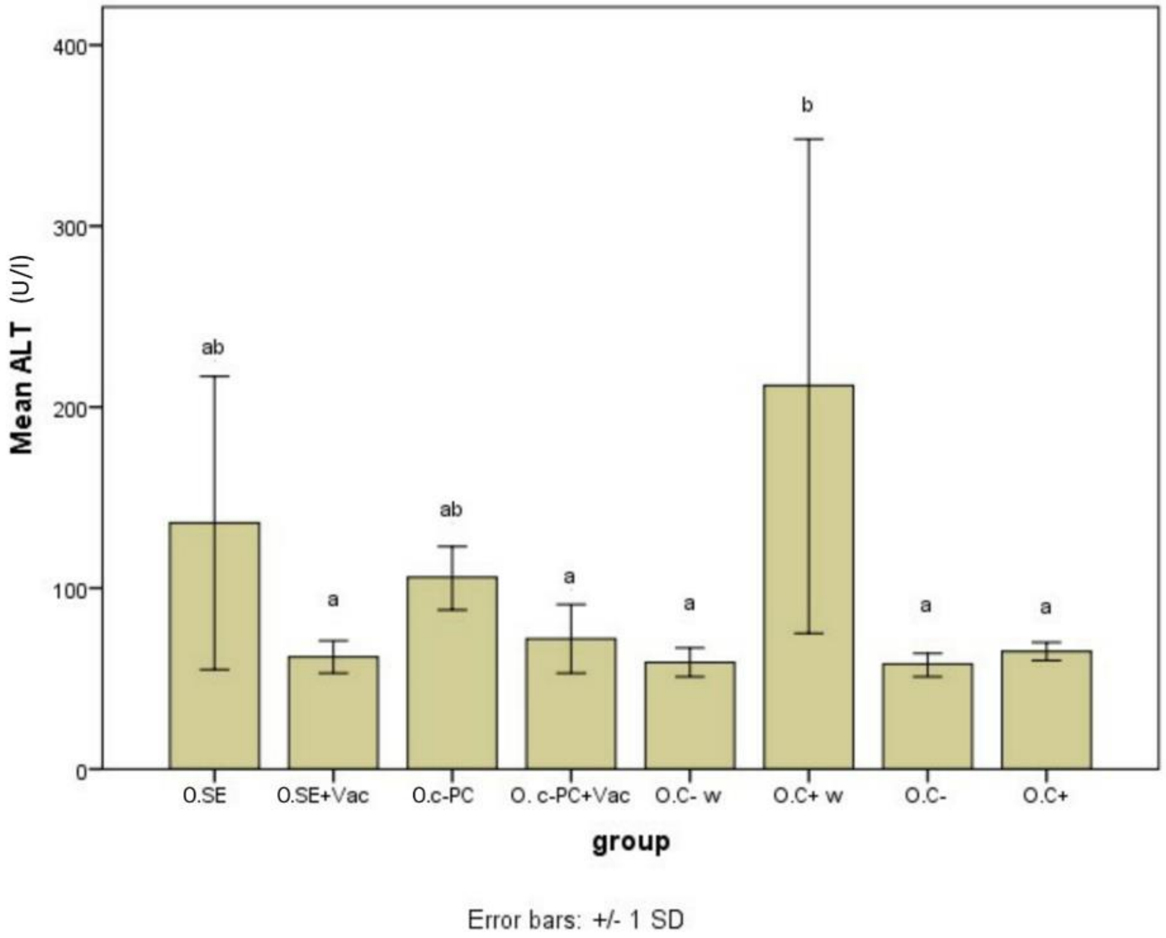

Appendix 7. Comparison of the average level of ALT changes in different groups receiving oral adjuvant (O.SE: 136.50, O.SE+Vac.:62.17, O.C-PC: 106.00, O.C-PC+Vac.: 72.50, O.C-w: 59.00, O.C+w:212.00, O.C-: 58.00 & O.C+:65.33). Although, the treatments have been categorized into 2main groups (a & b), significant differences are classified as groups: a, ab & b. While a groups (a & ab) and b groups (b & ab) have no significant difference in their own groups.

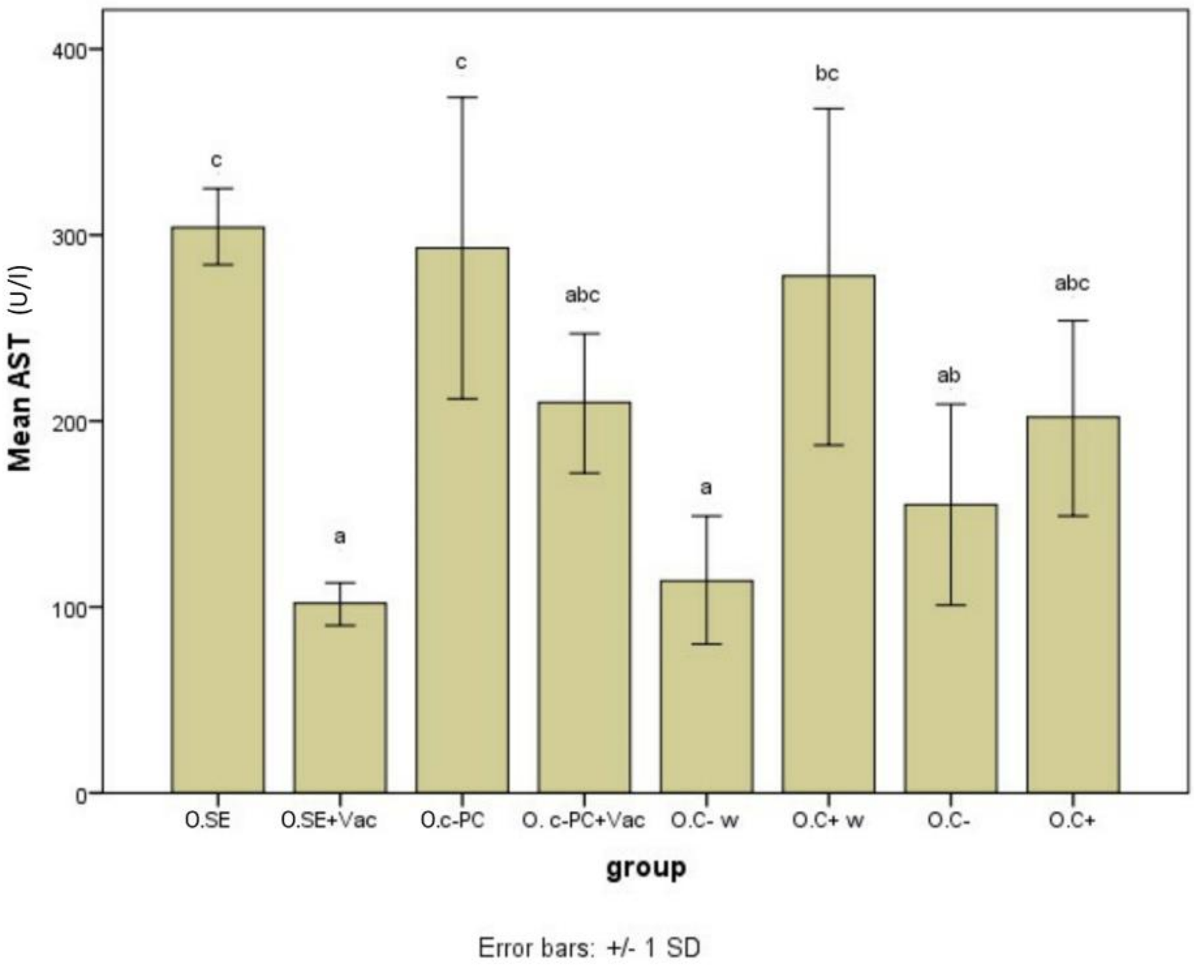

Appendix 8. Comparison of the average level of AST changes in different groups receiving oral adjuvant (O.SE: 304.75, O.SE +Vac.: 102.17, O.C-PC: 293.50, O.C-PC +Vac.: 210.00, O.C-w: 114.67, O.C +w: 278.33, O.C-: 155.67 & O.C+: 202.33). Although, the treatments have been categorized into 3 main groups (a, b & c), significant differences are classified as groups: a, ab, bc, abc & c. While a groups (a, ab & abc), b groups (bc & abc) and c groups (c, bc & abc) have no significant difference in their own groups.
